# Diagnosis and management of intraoperative thyroid storm in a child with undiagnosed Graves’ disease: a case report

**DOI:** 10.1186/s40981-022-00543-2

**Published:** 2022-07-16

**Authors:** Amanda W. Schaefer, Pedro J. Solorzano, Ana C. Mavarez, Gustavo M. Muñoz-Monaco

**Affiliations:** 1 Medical College of Georgia at Augusta University, Augusta, GA USA; 2 Department of Anesthesiology and Perioperative Medicine, Medical College of Georgia at Augusta University, Augusta, GA USA; 3 Department of Anesthesiology and Perioperative Medicine, Pediatric Anesthesiology Division, Children’s Hospital of Georgia, Medical College of Georgia at Augusta University, Augusta, GA USA

**Keywords:** Thyroid storm, Thyrotoxicosis, Graves’ disease, Pediatrics

## Abstract

**Background:**

Thyroid storm is a life-threatening manifestation of thyrotoxicosis and presents with fever, diaphoresis, tachycardia, hypertension, and widened pulse pressure.

**Case presentation:**

We present a case of intraoperative thyroid storm in a 12-year-old female undergoing posterior spinal fusion. Despite adequate depth of anesthesia and analgesia, the patient was persistently tachycardic and hypertensive. The surgical procedure was uneventful. A thyroid panel drawn immediately after surgery showed undetectable thyroid stimulating hormone (TSH) and high free thyroxine (T4) consistent with thyroid storm.

**Conclusions:**

Intraoperative thyroid storm in a pediatric patient is extremely rare with nonspecific clinical symptoms. Low to undetectable TSH and elevated free T4 is diagnostic.

## Background

Thyroid storm, an acute, life-threatening hypermetabolic state caused by excess circulating thyroid hormones, can be precipitated by surgery, infection, or trauma. Presentation includes fever, tachycardia, hypertension, and widened pulse pressure [1]. In a pediatric patient, such presentation intraoperatively might give the impression of a more common cause of intraoperative hyperthermia, tachycardia, and hypertension such as malignant hyperthermia [2], neuroleptic malignant syndrome [3], or pheochromocytoma crisis [4] since provoked or precipitated pediatric thyrotoxicosis has no documented incidence rate and could be easily misdiagnosed [5]. Graves’ disease, although the most common cause of hyperthyroidism in adults and children, is very uncommon in pediatric patients with an incidence of about 1 in 10,000, and pediatric thyroid storm is very rare [6]. Swift recognition of thyroid storm and initiation of treatment must be made to prevent further decompensation and restore homeostasis [7]. The patient’s parent provided written informed consent for publication of this case report.

## Case presentation

An otherwise healthy 45 kg 12-year-old Hispanic female with a past medical history significant for idiopathic progressive thoracic scoliosis with double curve of the thoracic spine presented for T2–T12 posterior spinal fusion in December 2020. She had no previous hyperthyroid symptoms and no family history of any thyroid disorders or autoimmune conditions. She had no prior surgical history.

On the day of the procedure, the patient’s vital signs as follows were significant for tachycardia and hypertension during the preanesthesia evaluation: temperature 36.5 °C, pulse 169 bpm, respiratory rate 22 breaths/min, and blood pressure 143/89 mmHg. Her heart rate continued to elevate to the 180s. Although she initially appeared calm, she reported that she was very nervous and cried upon questioning. She also had a fine tremor, which in combination with her tachycardia was attributed to severe anxiety. She was given midazolam, and her heart rate improved to low 160s.

The patient was induced with sevoflurane and 50% nitrous oxide via mask, then given fentanyl (50 mcg), rocuronium (30 mg), propofol bolus (100 mg), and intubated with a 6-mm internal diameter cuffed endotracheal tube. At the start of the case, she was given cefazolin (1500 mg) and dexamethasone (4 mg), and a nasogastric tube was placed. After induction her anesthesia was maintained with sevoflurane and propofol, and remifentanil infusion and fentanyl boluses were given for intraoperative pain management. Tranexamic acid infusion was started for intraoperative bleeding prophylaxis. Sugammadex (100 mg) was used to reverse neuromuscular blockade for neuromonitoring. An initial arterial blood gas (ABG) at the start of surgery showed a pH of 7.3, partial pressure of carbon dioxide (PaCO_2_) 40 mmHg, partial pressure of oxygen (PaO_2_) 362 mmHg, base excess of − 2, and bicarbonate (HCO_3_^−^) 23 mEq/L on 100% fraction of inspired oxygen (FiO_2_).

Despite adequate depth of anesthesia and analgesia according to neuromonitoring, the patient remained tachycardic to the 160s with pulse remaining above 110 bpm throughout the case. She was hypertensive for most of the case with systolic blood pressures ranging from 100s to 140s mmHg and diastolic blood pressures ranging from 40s to 70s mmHg. About 3.5 h after induction, her temperature exceeded 38 °C. The IV warmer was turned off and the Bair Hugger blanket was turned to ambient air to cool the patient. The patient reached a max temperature of 38.4 °C. Because her fever did not rapidly progress and decreased after withholding warming measures and PaCO_2_ was stable on subsequent ABGs, malignant hyperthermia was unlikely, so dantrolene was not administered. Despite the patient’s tachycardia and hypertension, she was considered stable enough for the continuation of surgery, which was completed without complications. The patient received midazolam (2 mg), ondansetron (4 mg), and morphine (2 mg) prior to emergence from anesthesia. The patient was extubated in the operating room without any complications.

The patient was then transferred to the PICU with tachycardia and mild hypertension: temperature 36.9 °C, pulse 140 bpm, respiratory rate 28 breaths/min, and blood pressure 128/64 mmHg. A complete blood count and electrolyte panel drawn immediately after surgery were within normal limits. A thyroid panel was significant for undetectable thyroid stimulation hormone (TSH), high free thyroxine (T4) (4.46 ng/dL), high triiodothyronine (T3) (3.16 ng/mL), and high free T3 (13.6 pg/mL) and was consistent with thyroid storm. An electrocardiogram did not indicate any underlying cardiovascular dysfunction such as atrial fibrillation or heart failure. Propylthiouracil, iodine, hydrocortisone, and esmolol infusion were initiated. Endocrinology was consulted, and the patient was diagnosed with Graves’ disease. She was transitioned from propylthiouracil to methimazole and from esmolol to propranolol. She was discharged on postoperative day 3 on methimazole and propranolol.

The patient eventually underwent total thyroidectomy. Her sister was also subsequently diagnosed with Graves’ disease, suggesting the presentation of Graves’ disease may be due to genetic factors.

## Discussion

Our patient’s presentation of intraoperative tachycardia and hypertension has a wide differential diagnosis where usually thyroid storm would be unexpected. Thyroid storm in children with no prior thyroid disease or precipitating event is rare with an incidence of only 1 in 1,000,000 in children and 3 in 100,000 in adolescents. Surgery, trauma, burns, medications such as lithium and amiodarone, exposure to iodine or contrast agents, and direct thyroid trauma can precipitate thyroid storm. Autoimmune thyroid disease including Graves’ disease, which accounts for 95% of pediatric hyperthyroidism [1], is associated with several single nucleotide polymorphisms in immune-regulating genes including FOXP3, CD25,CD40, CTLA-4, the HLA genes, and PTPN22, which may be the case for our patient since her sister is also affected [8]. Because hyperthyroidism affects nearly all organ systems with nonspecific clinical symptoms, diagnosis can be difficult. Signs of thyroid storm include hyperthermia, tachycardia, hypertension with widened pulse pressure, arrhythmias, and congestive heart failure. Symptoms can progress to include severe nausea, vomiting, diarrhea, agitation and anxiety, delirium, seizures, and coma. Lab values are significant for very low to undetectable TSH and high free T4 [9].

Presentation may be similar to malignant hyperthermia, neuroleptic malignant syndrome, or pheochromocytoma (Table 1). Malignant hyperthermia is a rare, inherited metabolic disorder that can be initiated by halogenated volatile anesthetic agents or succinylcholine [10], and it presents with rapidly increasing body temperature, muscle rigidity, masseter spasm, increasing end tidal carbon dioxide (CO_2_), and in late stages, acidosis and myoglobinuria.^2^ Neuroleptic malignant syndrome is precipitated by antipsychotics, antiemetics, or decrease or withdrawal of anti-parkinsonian drugs and presents with hyperthermia, hypertension, tachycardia, altered mental status, extrapyramidal symptoms, muscle rigidity, and rhabdomyolysis with symptoms developing over a 24- to 72-h period [3]. Lastly, pheochromocytomas are rare neuroendocrine tumors that typically arise in the adrenal medulla and secrete catecholamines, which cause episodes of hypertension, tachycardia, headaches, and diaphoresis, and they can result in intraoperative hypertensive crisis if undiagnosed pre-surgery [4].

**Table 1:** Differential diagnosis for intraoperative tachycardia and hypertension

	Thyroid storm	Malignant hyperthermia	Neuroleptic malignant syndrome	Pheochromocytoma
**Underlying pathology**	• **Severe state of thyrotoxicosis** caused by excess circulating thyroid hormones	• Inherited metabolic disorder **initiated by halogenated volatile anesthetic agents** or succinylcholine	• **Precipitated by typical or atypical antipsychotics**, antiemetics, or change in anti-parkinsonian drugs	• Rare **neuroendocrine tumor** of the adrenal medulla • Secretes **catecholamines**
**Presentation**	• **Hyperthermia**^**a**^ • **Tachycardia**^**a**^ • **Hypertension**^**a**^ with widened pulse pressure • Arrythmias • Congestive heart failure	• **Hyperthermia**^**a**^ • **Tachycardia**^**a**^ • **Hypertension**^**a**^ • Muscle rigidity • Masseter spasm • Increasing end-tidal CO_2_ • Metabolic acidosis • Myoglobinuria	• **Hyperthermia**^**a**^ • **Tachycardia**^**a**^ • **Hypertension**^**a**^ • Altered mental status • Extrapyramidal symptoms • Muscle rigidity • Rhabdomyolysis	• **Hypertension**^**a**^ • **Tachycardia**^**a**^ • Headaches • Diaphoresis • Intraoperative hypertensive crisis
**Intraoperative diagnosis**	• Thyroid panel with **low to undetectable TSH** and **high free T4**	• Clinical diagnosis during acute event based on patient presentation • Can later do susceptibility testing	• Clinical diagnosis based on patient presentation and current medications • **Elevated creatine kinase**	• Clinical suspicion based on **hypertensive crisis** and patient history • **Elevated plasma free metanephrines** • Post-op imaging
**Immediate treatment plans**	• Intraoperatively manage hemodynamics (beta blockers, cooling blankets, vasodilators)	• Discontinue halogenated agent • Administer dantrolene • Provide “clean” source of oxygen	• Discontinue causative agent (dopamine antagonists) and stop potential contributing agents • Provide supportive care to maintain cardiopulmonary stability and euvolemia	• Initiate alpha adrenergic blockade (phentolamine or phenoxybenzamine) • Treat hypertension and arrythmias • Discontinue surgery if possible

*Abbreviations*: *CO*_*2*_ carbon dioxide, *T4* thyroxine, *TSH* thyroid stimulating hormone

^a^Similar symptoms across conditions

Our patient had an elevated temperature likely due to warming blankets, no masseter spasm, and normal end tidal CO_2_, ruling out malignant hyperthermia. She had no history of medication use related to neuroleptic malignant syndrome. Although she was hypertensive intraoperatively, her blood pressure was not as elevated as would be expected in a hypertensive crisis due to pheochromocytoma, and she had no history of episodic headache, tachycardia, and diaphoresis prior to surgery. Considering this differential, our patient’s persistent tachycardia and hypertension with widened pulse pressure along with her thyroid panel indicate thyroid storm.

We performed a PubMed search using the following key terms: (“thyroid storm” or “thyrotoxicosis”) and (“child” or “pediatric”) and “intraoperative”. The results returned seven articles, of which, only two described cases of intraoperative thyroid storm in a child. In one case, a 5-year-old male, had an enlarged thyroid prior to surgery that was missed [7]. In the other case, a 15-year-old male underwent surgery following trauma, but after further questioning, he reported having previous signs of hyperthyroidism prior to the trauma [11]. In contrast, our patient denied any symptoms of hyperthyroidism such as tachycardia, fatigue, anxiety, or inability to gain weight prior to surgery, and she had no previous family history of thyroid problems. Our patient’s diagnosis was challenging to identify since the only signs of hyperthyroidism prior to surgery were her tachycardia, hypertension, and fine tremor during the preanethesia evaluation, which were attributed to severe anxiety. Further questioning and observation preoperatively may have revealed additional signs and symptoms of hyperthyroidism which would have prompted a thyroid panel collection, allowing for earlier diagnosis and reducing risk of progression to thyroid storm.

Thyroid storm if left untreated is associated with significant mortality up to 90% in adults with likely similar outcomes in pediatric patients [5]. Intraoperative treatment is mostly supportive, including beta blockers and vasodilators as needed to manage hemodynamics and cooling blankets for hyperthermia. A thionamide, beta blocker, glucocorticoid, and iodine in combination are recommended. A thionamide inhibits new thyroid hormone synthesis, where propylthiouracil is preferred due to its additional inhibition of the conversion of free T4 to the more potent T3. Beta blockade inhibits adrenergic symptoms, and propranolol is preferred because it partially blocks peripheral conversion of T4 to T3. A glucocorticoid, usually hydrocortisone, prevents relative adrenal insufficiency and also blocks peripheral T4 to T3 conversion. Iodine, usually potassium iodide solution, prevents new hormone synthesis and additional thyroid hormone release [9].

In conclusion, intraoperative thyroid storm in pediatric patients is very rare, and many clinical symptoms are nonspecific, resulting in a broad differential diagnosis. Because thyroid storm in pediatric patients is normally lower on the differential, more common conditions must be identified quickly or ruled out based on patient presentation. A thyroid panel with low to undetectable TSH and elevated free T4 in the presence of hyperthermia, tachycardia, hypertension, and widened pulse pressure is diagnostic for thyroid storm. Initiation of treatment to correct the hyperthyroidism, restore homeostasis, and treat decompensating factors is vital to reduce morbidity and mortality.

## Data Availability

Not applicable

## Abbreviations

ABG: Arterial blood gas
CO_2_: Carbon dioxide
FiO_2_: Fraction of inspired oxygen
HCO_3_^-^: Bicarbonate
PaCO_2_: Partial pressure of carbon dioxide
PaO_2_: Partial pressure of oxygen
T3: Triiodothyronine
T4: Thyroxine
TSH: Thyroid stimulating hormone
